# Timing variability of sensorimotor integration during vocalization in individuals who stutter

**DOI:** 10.1038/s41598-018-34517-1

**Published:** 2018-11-05

**Authors:** Anastasia G. Sares, Mickael L. D. Deroche, Douglas M. Shiller, Vincent L. Gracco

**Affiliations:** 1 Integrated Program in Neuroscience and School of Communication Sciences and Disorders, McGill University, Montréal, QC Canada; 2 École d’orthophonie et d’audiologie, Université de Montréal, Montréal, QC Canada; 3 Haskins Laboratories, New Haven, CT USA; 4 Centre for Research on Brain, Language, and Music, McGill University, Montréal, QC Canada

## Abstract

Persistent developmental stuttering affects close to 1% of adults and is thought to be a problem of sensorimotor integration. Previous research has demonstrated that individuals who stutter respond differently to changes in their auditory feedback while speaking. Here we explore a number of changes that accompany alterations in the feedback of pitch during vocal production. Participants sustained the vowel /a/ while hearing on-line feedback of their own voice through headphones. In some trials, feedback was briefly shifted up or down by 100 cents to simulate a vocal production error. As previously shown, participants compensated for the auditory pitch change by altering their vocal production in the opposite direction of the shift. The average compensatory response was smaller for adults who stuttered than for adult controls. Detailed analyses revealed that adults who stuttered had fewer trials with a robust corrective response, and that within the trials showing compensation, the timing of their responses was more variable. These results support the idea that dysfunctional sensorimotor integration in stuttering is characterized by timing variability, reflecting reduced coupling of the auditory and speech motor systems.

## Introduction

Stuttering is a neurodevelopmental disorder affecting approximately 1% of the adult population; it consists of undesired repetitions, prolongations, and blockages of speech sounds, syllables, and words^1^. The cause of stuttering is unclear, but the disorder is associated with, among other factors, a problem with sensorimotor integration^2^. Sensorimotor integration for speech involves the coupling, through feedback and feedforward processes, of sensory information and motor commands during self-generated movement to produce appropriate, goal directed responses. The importance of such coupling between sensory and motor processes has been shown in some classic studies employing visual prisms to change the coordinate space for reaching^3,4^. Changes in sensory feedback induce a rapid adjustment in the motor commands to rearrange the sensorimotor coordinate space. Interestingly, when the sensory modification is applied during passive movement, behavior does not adapt, highlighting the importance of motor and sensory coupling during active movement^5^. These early studies clearly illustrate the importance of sensorimotor coupling in developing and maintaining goal-oriented motor actions.

For speech production, studies using alterations in sensory feedback have demonstrated a similarly strong coupling between sensory and motor processes^6–14^. In studies using auditory feedback manipulations, a participant speaks into a microphone and their own voice is presented back to them through headphones in real time. Feedback to the headphones is manipulated to simulate a production error, changing aspects such as the pitch of the voice or the resonant structure of the speech signal (for example, shifting the heard sound from an /ɛ/ to an /i/). In response to the manipulation, the participants reflexively change their output to correct the discrepancy^15,16^. If the manipulation is stable and maintained over successive trials, an adaptive process is engaged and a change in sensory and motor representations takes place^12–14^. In contrast, if the manipulation is intermittent or unpredictable, an on-line correction process will counteract the errors, but the sensory and motor representations do not adapt to any “new normal”. Thus, compensatory responses can be used to assess the properties of the real-time control system. These compensatory responses to unpredictable changes in sensory feedback are the focus of the current study.

Studies of typically developed adults have focused on a number of properties of the speech motor control system inferred from the dynamics of the compensation response. Alterations to an unpredictable somatosensory or auditory feedback signal have been used to evaluate the gain (or sensitivity) of the system^6,11,16^, the latency of the response^6,15,16^, or the precision of the system, estimated through the variability of the response^6,7,15,17–20^. Some studies using altered auditory feedback have observed different categories of responses, including an “opposing response”— the expected compensatory response that goes in the opposite direction of the perturbation and counteracts the induced error—and a “following response”— a less-understood response that goes in the same direction as the auditory perturbation, accentuating the induced error rather than counteracting it^16,19,21–23^.

Similar studies with adults who stutter have reported reduced compensatory responses to auditory feedback manipulations^24–26^, which would seem to indicate a problem with modulating the output gain. Cai and colleagues^24^ examined compensation of F1 (the first resonant frequency, or formant, of a speech signal that helps define vowel quality) during perturbations of the vowel /ɛ/, and found that the response was attenuated in individuals who stuttered. In terms of pitch compensation, Bauer and colleagues^25^ found that responses to pitch shifts occurred later in time for people who stuttered than a control group, especially for small pitch shifts. They did not find any difference in the magnitude of pitch compensation at the level of individual trials. However, with only 4 subjects per group, these findings were preliminary and in need of replication. In 2012, Loucks, Chon, & Han tested a larger sample^26^, and showed that in the average opposing response, people who stuttered compensated less for pitch shifts than controls, and again exhibited a very slight delay. However, these results were largely descriptive. Throughout these studies, it seems that there is a tendency for people who stutter to have slightly fewer compensating trials, and to have a slightly delayed response, but the magnitude of compensation is not in fact compromised during those trials in which a compensatory response is observed. The first aim of the present study is to replicate and examine these findings in more detail.

Surprisingly, none of these previous studies have looked at the variability in the timing of the pitch compensation response, despite the fact that timing variability is a signature of stuttering. Earlier studies attempting to examine vocal pitch differences in the speech of people who stutter found differences in duration variability instead^27,28^ (but see Healey, 1982 for different results)^29^. More recently, evidence has accumulated that timing variability is increased in stuttering^30–33^, and that stuttering behavior may be related to a general temporal processing limitation^34–40^. Building on behavioral evidence of timing as a disordered control variable in indivuduals who stutter, studies using electroencephalography (EEG) have have examined dysfunctional neural coherence as a significant explanatory property associated with the speech of individuals who stutter^41,42^. As a result, timing variability in the compensatory response to pitch alterations in individuals who stutter was a major focus of the current study.

Here we explore in detail the amplitude and variability of the pitch-shift compensation response in individuals who stutter. Based on research from previous perturbation studies, individuals who stutter are assumed to produce smaller average compensation curves^43–45^. Yet this reduced compensation curve could result from averaging trials with a more generalized timing problem^46^ in which timing variability stems from an inability to integrate sensory input with motor output in an optimal manner. Thus, we expect this smaller compensation curve to be the result of variable timing in individual trials, rather than a simple decrease in their magnitude.

## Methods

### Participants

Nineteen adult controls (AC) and nineteen adults who self-identified as having a stutter (AS) from ages 18–51 (10 women, 9 men per group) participated. None had neurological, speech or language problems. Control participants were matched in sex and age within 5 years of a stuttering participant. All stuttering participants except two reported previous diagnosis by a speech-language pathologist; all except three had undergone some form of speech therapy. A trained speech-language pathologist specializing in stuttering, blinded to each participant’s classification, was given 10-minute videos of natural speech productions from the testing session (combining of reading, image description, and conversation), and was asked to classify them as AC or AS, and rate the severity of each stuttering participant according to the Stuttering Severity Instrument, 4th edition (SSI-4)^47^. In addition, every stuttering participant self-rated their stuttering severity on a scale of 1 to 9 reflecting their experience with speech in daily life^48,49^.

The two types of severity ratings (speech-language pathologist and self-rated) were highly correlated (r = 0.7647, p = 0.0001), consistent with previous studies; however, the speech-language pathologist allocated five individuals with a stutter to the control group, and four controls to the stuttering group. The five misclassified stuttering participants had low severity ratings (mean self-rating of 3+/−0.94; range of 2–4.5); those classified as stuttering had higher severity (mean self-rating of 4.49+/−1.58; range of 2–7.5). Finally, the speech-language pathologist identified one participant as having characteristics of neurogenic stuttering. To be conservative, we excluded all participants who were misclassified and the participant with the neurogenic stutter, thus the data presented in this study are from 15 AC participants (5 male, 10 female) and 13 AS participants (6 male, 7 female). They ranged in age from 18–51 years (mean 28+/−10 for the AS, mean 27+/−9 for AC). This study was approved by the McGill Faculty of Medicine Institutional Review Board in accordance with principles expressed in the Declaration of Helsinki; informed written consent was obtained from participants prior to their involvement in the project.

### Procedure

Participants produced 74 vocalizations of the vowel /a/ (“ah”) for approximately 1.6 seconds while hearing their own voice through headphones. Prior to beginning the task, the experimenter provided 1–2 example vocalizations and a small number of the participant’s preliminary vocalizations were used to adjust the output signal level to a comfortable volume. During the 74 production trials, participants were instructed to vocalize for a precise length of time, receiving feedback on whether or not they were close to the target duration (durations of 1.4–1.8 s were considered correct). They were not explicitly told to match any pitch or “sing” with a constant pitch. Further, participants were not informed in advance about the pitch shifts to make compensation response as naturalistic as possible. For 24 of the 74 trials, the fundamental frequency of the voice, as heard through the headphones, was shifted upward 100 cents (cents being a logarithmic scale for pitch used in music instruments that better corresponds to human pitch perception). The shift had a duration of 500 ms (onset varied between 350 and 800 ms to make it less predictable). In another 24 trials, the pitch was shifted down by the same amount. In the remaining 26 trials, no pitch shift was applied. Up, down, and no shift trials were randomized.

The voice manipulation was carried out in near-real time by capturing the voice via microphone and using software (Audapter)^24,50,51^ to extract and manipulate the fundamental frequency (F0). Feedback was fed to the individual via Sony MDR-ZX300 over-the-ear headphones with less than 25 ms delay, and mixed with pink noise to reduce perception of the unmodulated air- and bone-conducted acoustic signal. Pink noise measured approximately 64 to 69 dB, and participants’ vocal feedback playing through the headphones was approximately 74 to 78 dB.

The procedure involved the production of the isolated vowel/a/, which is a low complexity utterance resulting in very few dysfluencies. The perturbation was applied following 350–800 ms of stable vocalization. In addition, the program controlling the experiment automatically repeated any trial with a break in the sound (or no vocalization at all). In other words, if any dysfluencies occurred, the trial would be repeated as many times as needed until it could run smoothly. The mean number of repeated trials per subject was 7.8 for the AC group and 4.5 for the AS group, with one outlier in the control group (31 repetitions). Finally, all accepted trials were verified by one of the authors (M.D.) to ensure they contained continuous vocalization. None were rejected for dysfluencies.

### Analysis

In a preliminary step, the concatenated vocal production signal over all trials was passed through the PSOLA pitch detection algorithm in PRAAT^52^, which gave frequency estimates (in Hz) every 10 ms. The distribution of pitches over all trials was acquired, and the primary mode of this distribution was identified for each participant as their characteristic voice pitch. Subsequent pitch analysis was restricted to +/−8 semitones around this characteristic pitch in order to prevent octave errors (97% of pitches were unaffected by this).

Next, a pitch trace (10 ms steps) was obtained for each trial, again using the PSOLA algorithm. The data were imported into MATLAB (R2015a)^53^. Trials were aligned at the onset of the perturbation (or, for control trials, a randomly-selected point where a perturbation could have occurred). Though participants always vocalized for at least 350 ms before the perturbation, 300 ms before the perturbation onset was taken as the trial baseline to avoid the first 50 ms, where pitch was not stable. Pitch traces of individual trials were expressed in cents relative to the F0 at the beginning of the perturbation. This was to control for individual differences in F0 as well as drift over the course of the trial or experiment. The following equation (1) was used to convert hertz to cents:

1 $$cents=1200\ast log\,2(freq/{freq}_{PertOnset}\,)$$

### Normalizing and categorizing trials

**Figure 1:**
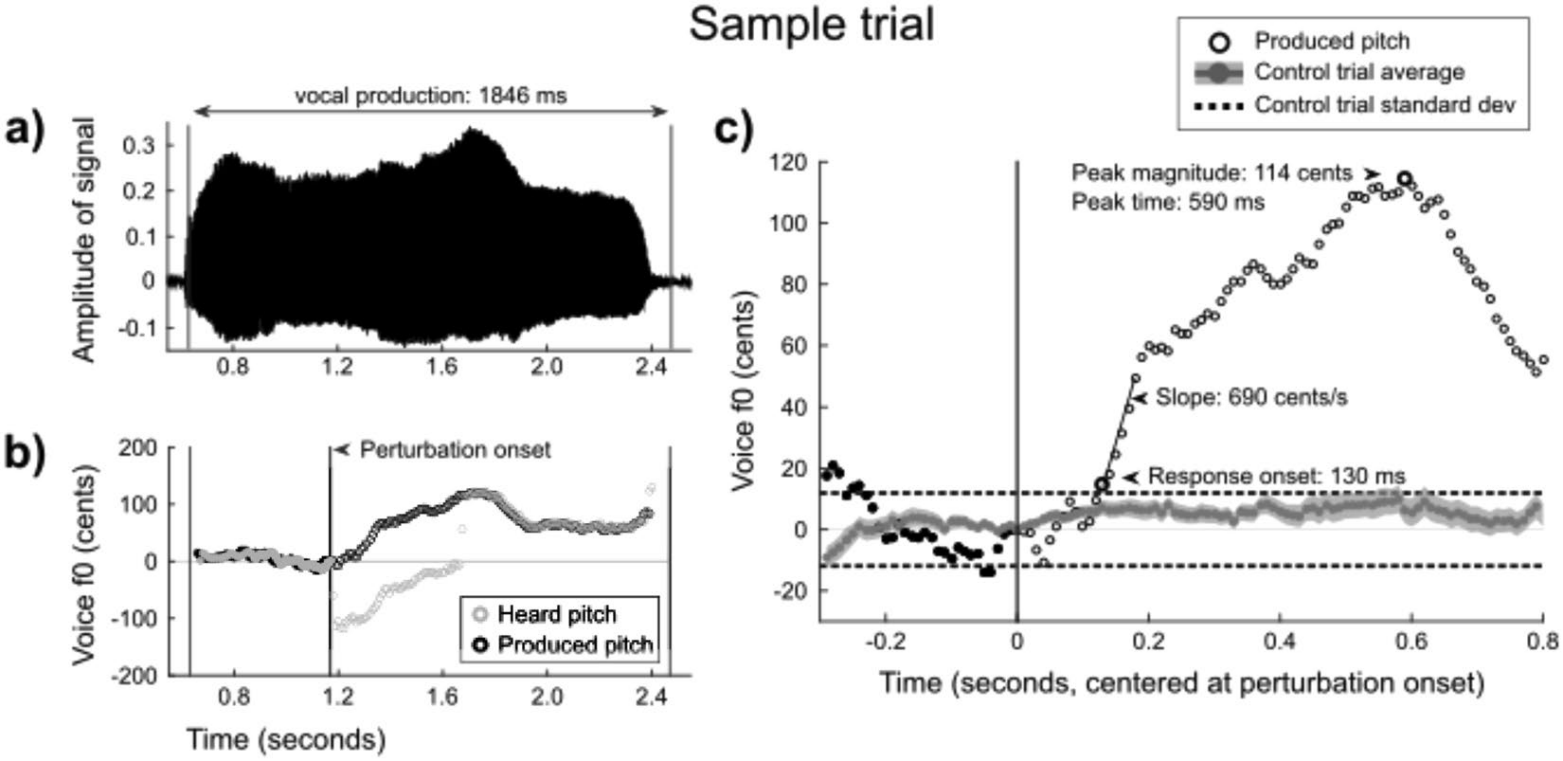
A sample trial. (**a**) vowel waveform; (**b**) pitch trace for this waveform showing the participant’s vocal output (black) and what they heard (light gray). In this case, there was a down-shift so that the heard pitch was 100 cents below the produced pitch over a 500-ms duration; (**c**) the participant’s mean control trace (gray area-fill) was subtracted from the vocal output of each trial to create a normalized response (black). Dotted lines represent the +/−1 standard deviation of the control trials. All pitch traces are in cents, centered at the frequency that was being produced at the shift onset. In this trial, response onset is identified at 130 ms with an onset slope of 690 cents/s (F0 increased by 34.5 cents over the next 50 ms), and the peak time is at 590 ms, with a peak magnitude of 114 cents. Note: Time is measured from the beginning of the trial in (**a**,**b**), and realigned to the shift onset in (**c**). Filled circles in (**b**) and (**c**) represent the baseline period for that trial.

For some participants, pitch tended to rise or fall over the course of a trial, so control trials were first averaged together to obtain a characteristic trace for each participant and a standard deviation (SD) to represent that participant’s pitch variability, determined from the 300 ms baseline period during control trials. Each trial was then normalized by subtracting the characteristic pitch trace for each subject. After this, responses to a shifted trial were classified as “opposing”, “following” or “no change”: opposing responses go in the opposite direction of the perturbation (e.g. a positive-going response for a −100 cent perturbation), and following responses go in the same direction as the perturbation. To categorize the type of responses that a given trial represented, we ran a peak detection algorithm on the pitch trace, from the onset of perturbation, with a few constraints: 1) peak magnitudes had to be greater than +/−1 SD from the zero point, and 2) peak times could not be selected from 0–50 ms (based on possible onset times reported in a previous pitch-perturbation study)^16^ or 780–800 ms (end of the trace) of the post-perturbation period. Peaks that did not exceed +/−1 SD were labeled “no change”. The onset time was identified as the beginning of the first 50 ms window where vocal pitch was entirely above 1 SD from baseline in the same direction as the peak. If no peak had been found, no onset was searched for. We then defined the onset slope as the slope of the pitch trace during this 50 ms window. For a sample trial, see Fig. 1.

A few trials were eliminated because the PSOLA algorithm (PRAAT) failed to detect a consistent pitch (due to creaky voice, for example). Most participants had 3 or fewer trials eliminated, except for one control participant who had 11 trials eliminated.

### Time series: Average response

In addition to the timepoint-by-timepoint representation in the figures, we calculated the area underneath the curve for each subject and condition, entering the results into a 2-way ANOVA.

### Number of opposing trials

We counted opposing, following, and no-change responses for each individual and submitted the results to an ANOVA. Since the three categories are exactly collinear, we only included “following” and “opposing” categories, along with two trial types: up-shifts and down-shifts, yielding a 2 × 2 × 2 design (group, shift type, and response type). We performed a Pearson correlation between the number of “opposing” responses and stuttering severity within the AS group.

### Magnitude of opposing responses

To investigate whether the magnitude of the “opposing” responses was attenuated for AS, we performed a two-way ANOVA (group & shift type) on the area underneath each participant’s average curve for responses identified as opposing.

### Timing variability of opposing responses

We looked at four mean measures, considering only “opposing” responses: (1) onset time, (2) onset slope, (3) peak time, and (4) peak magnitude, performing ANOVAs for each with a 2 × 2 design (group & shift type). We did the same thing for two measures of timing variability: (1) standard deviation of onset time and (2) standard deviation of peak time. We performed Pearson correlations between the standard deviations of onset/peak time and stuttering severity. Finally, to see whether the variability of onset/peak time was related to the average response, we correlated the standard deviation of peak time with the peak magnitude of subjects’ overall curves (which includes opposing and no-change responses), and the area under the overall curves.

## Results

All results from Student’s t-tests are 2-tailed. The mode (and standard deviation) of vocal pitch for the AC group was, on average, 182 Hz (54 Hz); for the AS group it was 166 Hz (57 Hz) (t(26) = 0.76, p = 0.456 [n.s.]). Pitch variability over the 300 ms baseline of control trials (standard deviations, from which the classification threshold was determined), was on average 19.9 cents (SD across subjects = 6.4 cents) for AC and 21.4 cents (SD across subjects = 7.8 cents) for AS (t(26) = −0.53, p = 0.599 [n.s.]). Thus, neither baseline F0 nor F0 variability differed between groups.

### Raw responses

In the raw responses (i.e. responses to the perturbation before subtracting the control traces), we observed an overall pattern of compensation to the pitch perturbations, as documented in previous literature. The compensation pattern could be seen in the traces of individual participants, but there was a large amount of inter-individual variability, with some participants showing more compensation to down-shifts than up-shifts, and vice versa. When compared to no-shift trials, controls as a group displayed a strong response to shifted pitch in both directions, from roughly 140 ms to the end of the trial. Participants with a stutter also had responses to both shifts, but the responses seemed to have a more gradual onset.

We then normalized the response to up- and down-shifts by subtracting the characteristic pitch trace (average of control trials) of each participant individually (see methods section).

### Time Series: Differences in average response

**Figure 2:**
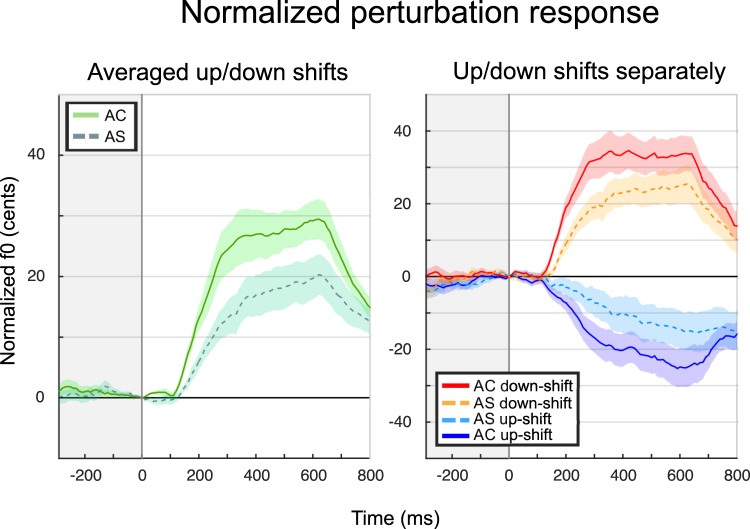
Averaged normalized pitch traces (before categorization into response types). Solid lines represent the AC group, and dotted lines represent AS. Shaded areas represent standard error of the mean. In the left panel, the absolute change in pitch for all shifts are presented, reflecting the average response independent of shift direction. In the right panel, the results for shift direction are presented with down-shift (red and orange), up-shift (blue and indigo).

As illustrated in Fig. 2 (left panel), considering up-shifts and down-shifts together and including all responses regardless of category, there seems to be a group difference in the response over a broad time window. Shift direction was found to have an influence on the response, as illustrated in the right panel.

For the area under the curve, there was a group effect (F(1,26) = 4.8, p = 0.038), an effect of direction (F(1,26) = 4.8, p = 0.037), and no interaction (F(1,26) < 0.1, p = 0.863). This is consistent with the differences we see in the traces. Thus, we replicate the finding that adults with a stutter have a smaller *average* response to pitch shifts, most notably in the presence of a down shift in F0 feedback. However, this result is potentially misleading, since as we will show, the groups had different numbers of “opposing”, “following”, and “no change” responses, as well as timing differences. In the following analyses, we address the different explanations for this apparent group difference.

### Number of “opposing”, “no-change” and “following” responses

**Figure 3:**
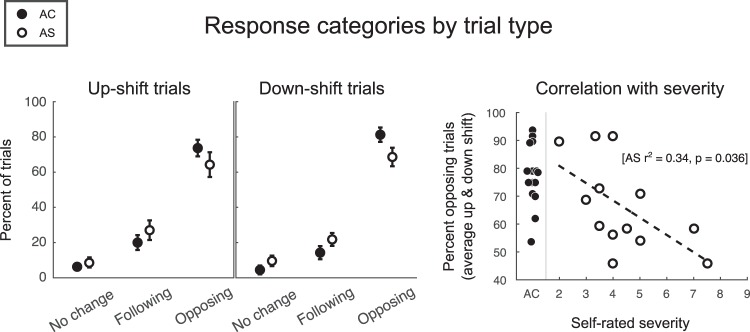
Response categories by trial type (left two panels), and correlation between severity and percent of opposing trials (right panel). Error bars represent standard error of the mean.

Fig. 3 shows the percentage of responses categorized as opposing, following and no change; roughly 10% of the responses displayed no significant change, 20% were “following” responses, and 70% were “opposing” responses. The pattern was similar for up-shift trials and down-shift trials. There was also a significant correlation between self-rated stuttering severity and the proportion of opposing trials (r^2^ = 0.34, p = 0.036).

The mixed-factor ANOVA revealed no main effect of group [F(1,26) = 1.5, p = 0.224], no main effect of trial type [F(1,26) < 0.1, p = 0.814], but a strong effect of category [F(1,26) = 132.2, p < 0.001]. The interaction between category and group was just significant [F(1,26) = 4.2, p = 0.050], and none of the other interactions (2- or 3-way) were significant [Fs(1,26) ≤ 1.4, ps ≥ 0.250]. The simple effects of group showed that AS obtained 7% more “following” responses than AC [F(1,26) = 3.0, p = 0.093; trending], and 11% fewer “opposing” responses than AC [F(1,26) = 4.5, p = 0.043; significant]. There was also a negative correlation with severity ratings, namely: the individuals with the most severe stuttering also demonstrated the lowest proportion of opposing responses. Stuttering severity accounted for 34% of the variance in the number of opposing trials within the AS group (r^2^ = 0.34, p = 0.036).

### Time series by trial type

**Figure 4:**
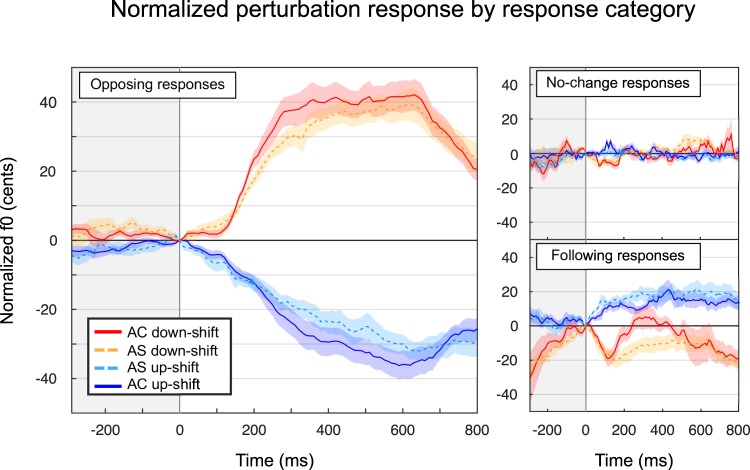
Perturbation responses by response category (opposing: left, no change: top right, following: bottom right). Solid lines indicate the AC group, and dotted lines represent AS. Shaded areas represent standard error of the mean.

For each participant, the responses categorized as “opposing” were pooled together and averaged for each participant, and then averaged across participants to provide the result presented in the left panel of Fig. 4. The top right panel is the averaged “no-change” response, which did not exceed variations of +/−10 cents, but contained a brief response around 150 ms when many trials were aggregated. The averaged “following” response (bottom right panel) exhibited variations and interesting differences between the two groups, particularly for downshifts, where AC participants exhibited a sudden rise in F0 around 150 ms and differed from AS between 180 and 430 ms, a pattern that seems largely reminiscent of the “opposing” response but appears to fall into the “following” category because of the descending trajectory within the initial 100 ms post perturbation onset.

Taking area under the curve for opposing responses only, there was no group effect (F(1,26) = 0.6, p = 0.432), but there was an effect of direction (F(1,26) = 8.3, p = 0.008), and no interaction (F(1,26) < 0.1, p = 0.890). This seems to indicate that clear opposing responses are not different between the two groups.

### Opposing trials: onset time, onset slope, peak time, and peak magnitude

Table 1 shows the results of the ANOVA analyses for the onset and peak of opposing responses. For trials categorized as “opposing” (roughly 70% of all trials), there was no effect of group on the mean values of onset time, onset slope, peak time, or peak magnitude. The compensation response to down-shift trials occurred slightly earlier and seemed to be more pronounced than the response to up-shift trials. Note that we obtain similar results if the threshold is reduced from 1 SD to 0 SD.

**Table 1:** ANOVA results for mean onset time, onset slope, peak time and peak slope (opposing responses only).

	Effect of Group	Effect of Direction	Group × Direction
Onset Time	F(1,26) = 1.0, p = 0.318	**F(1,26) = 11.5**, p = 0.002*	F(1,26) = 0.7, p = 0.421
Onset Slope	F(1,26) = 2.6, p = 0.122	**F(1,26) = 4.9**, p = 0.036*	F(1,26) < 0.1, p = 0.824
Peak Time	F(1,26) = 1.5, p = 0.234	**F(1,26) = 7.2**, p = 0.012*	F(1,26) < 0.1, p = 0.780
Peak Magnitude	F(1,26) = 1.0, p = 0.331	**F(1,26) = 4.2**, p = 0.049*	F(1,26) = 0.2, p = 0.675

Absolute values of onset slope and peak were used for a more representative comparison. Onset time: The onset of the compensation response occurred earlier for down-shifts than up-shifts. Onset slope: Slopes were steeper for down-shifts than up-shifts. Peak time: Peaks were earlier for down-shifts than up-shifts. Peak magnitude: Peaks tended to be slightly larger for down-shifts than up-shifts.

### Opposing trials: variability in onset and peak time

**Figure 5:**
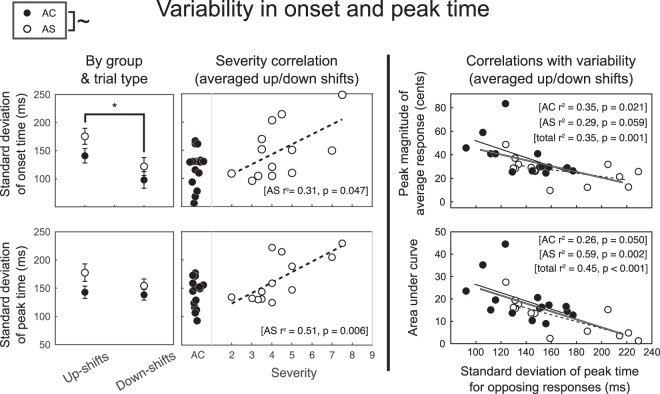
Group differences and severity correlations for variability (left and middle panels), and correlations of variability with the average response’s peak magnitude and AUC from 0 to 800 ms (right panel). Opposing responses only. Error bars represent standard error of the mean. The difference between AC and AS groups, marked with a tilde, was trending towards significance (onset time: p = 0.071; peak time: p = 0.054), and was corroborated by severity correlations that were significant (onset time: p = 0.047; peak time: p = 0.006).

Table 2 and Fig. 5 show results of onset time and peak time variability. For opposing trials, AS were more variable than AC in both onset time and peak time. This group difference was also corroborated by a strong relationship with severity ratings (onset timing: r^2^ = 0.31, p = 0.047; peak timing: r^2^ = 0.51, p = 0.006). However, it is important to bear in mind that these estimates of variance (onset and peak) were fairly correlated with each other for both AS and AC (AC: r^2^ = 0.22, p = 0.075; AS: r^2^ = 0.71, p < 0.001), and therefore do not represent two independent pieces of evidence for timing variability.

**Table 2:** ANOVA results and severity correlations for variability (standard deviation, SD) in onset time and peak time (opposing responses only).

	Effect of Group	Severity Correlation	Effect of Direction	Group x Direction
Onset Time Variability (SD)	**F(1,26) = 3.6**, **p = 0.071~**	***r2 = 0.31*** *,* p = 0.047*	**F(1,26) = 15.4**, p = 0.002*	F(1,26) = 0.2, p = 0.670
Peak Time Variability (SD)	**F(1,26) = 4.1**, **p = 0.054~**	***r2 = 0.51*** *,* p = 0.006*	F(1,26) = 1.7, p = 0.202	F(1,26) = 0.8, p = 0.385

Variability in onset time: There was a trend for AS as a group to be more variable than AC in the onset time of their response, corroborated by a significant correlation between onset time variability and stuttering severity. Up-shift trials also led to onset responses that were more variable in time than down-shift trials. Variability in peak time: There was a trend for AS as a group to be more variable than AC in the peak time of their response, corroborated by a significant correlation between peak time variability and stuttering severity.

## Discussion

We replicated a group difference in the ability to compensate for random perturbations in voice pitch, such that participants who stutter had a smaller averaged response. Upon closer examination of individual trials, it was revealed that people who stutter had fewer responses that could be reliably categorized as “opposing”, and that among their opposing responses, the timing of the response was more variable. Both the number of opposing trials and the timing variability were correlated with stuttering severity. *However, individual opposing trials did not differ reliably in peak compensation magnitude between the stuttering and control groups*, indicating that the difference between persons who stutter and the general population is likely due to variability of their responses. This pattern of results seems to suggest a noisy sensorimotor system rather than one with a reduced gain.

The key issue here is whether the magnitude or the variability of the response is more central to stuttering. Because we find that individuals who stutter do not differ in their response magnitude on “opposing” trials (neither in the time series nor the area under the curve), but they do differ when all trials are put together, it naturally leads one to the conclusion that the “following” and “no-change” trials are contributing to the difference. This interpretation is supported by a group difference in the proportion of opposing responses, and the strong correlation between the proportion of opposing responses and stuttering severity. But is a difference in the number of “following” trials indicative of a magnitude difference (fewer responses cross the 1 SD threshold because the responses are smaller overall), or is it indicative of increased variability (sometimes adults with a stutter will compensate, sometimes not)?

The categories of “opposing” and “following” have been proposed in the literature as possibly meaningful distinctions and indicative of positive versus negative feedback loops^16,19,21–23^. Yet the idea that opposing trials are somehow categorically different from other trials is undermined by the fact that following trials seem to have abnormal baseline trajectories that would make them more likely to be classified as “following” before the compensation response even has a chance to manifest (for a brief discussion of this idea, see Behroozmand *et al*.^21^). Furthermore, the “no change” trials and even some of the “following” trials seem to, in the aggregate, show hints of compensation behavior, but the individual peaks and onsets cannot be reliably identified for those trials and thus cannot be submitted to further analysis. At this point, one might reasonably assert that “following” and “no-change” trials are just sub-threshold (i.e. low-magnitude) compensation responses.

What the magnitude difference explanation does *not* account for are the differences in timing variability on opposing trials, which do suggest a variability explanation. These differences in timing variability are apparent when comparing groups, and are also related to stuttering severity.

To some extent, two effects contribute to an averaging issue (i.e. smoothing down the averaged response): (1) the relative number of trial types (opposing, following and no-change), and (2) the variability within opposing trials. However, the interpretation of the first effect is distinct from the second. The first effect seems to imply that AS participants do not behave in this task like AC participants (in opposing the pitch perturbation as often). The second effect, on the other hand, concerns the variance among trials that have all been categorized as “opposing”. Thus, even when the behavior is typical, there is still a timing problem in individuals who stutter. Ultimately, since both number of opposing trials and temporal variability are correlated with stuttering severity, it is not possible to tease these two explanations apart from the data presented here.

A simple reduction in vocal response magnitude, as suggested by earlier work, might stem from less reliance on auditory feedback as opposed to somatosensory, for example^43–45,54^, or it could be due to a reduced degree of flexibility in the feedback-correction system of people who stutter^25^. Indeed, Parkinson’s disease has often been contrasted with stuttering, in part because the former is treated by upregulating dopamine while the latter is sometimes treated by downregulating it^55^. Some research suggests that individuals with Parkinson’s disease show an increase in response magnitude compared to controls for a similar pitch perturbation paradigm^56,57^, making a the opposite pattern in stuttering a sensible result. However, previous research also supports the timing variability explanation, as people who stutter have more variable speech movements, even in childhood^30–32^, and adults may have increased variability in timing for manual as well as speech synchronization tasks^34–40^. Finally, people who stutter do not compensate for time-manipulated speech as well as controls^58^, and it is well known that fluency can temporarily be induced in people who stutter through the use of delayed auditory feedback^59,60^. Thus, it is reasonable to suggest that timing variability is a contributing factor in the different compensation response.

In the present study, the most robust difference between AS and AC groups was in measures of timing and timing variability in both the onset time and time of peak response. We suggest that these timing effects reflect a reduction in the strength of the coupling (or coordination) between the speech motor output and auditory feedback, consistent with previous findings of increased latency auditory evoked activity^61,62^. The perturbation is sensed and the magnitude of the adjustment is generally in line with the altered feedback. However, the timing variability results in an intermittent and presumably unpredictable delay in the response. The well-known observation of general slowness in the fluent speech of individuals who stutter (cf. Max, Caruso, & Gracco, 2003 for summary)^63^ may reflect an attempt to more fully integrate motor outflow with sensory feedback. Overall, we suggest that the variable timing between the auditory and motor systems reduces the coordination between them, and this reduced coupling leads to an unstable (or noisy) sensorimotor system. The instability leads to subtle variations in the fluent production of the speech of individuals who stutter and is a primary contributor to the increased dysfluency secondary to increased linguistic and/or cognitive demands^64^.

It is worth noting that this study looks only at pitch, which is primarily involved in suprasegmental aspects of communication (at least in non-tonal languages), whereas the overt behavior of stuttering itself deals mostly with repeated segments of speech. The fact that this timing variability is present even in a “suprasegmental” feature may indicate that this is a more general auditory-motor issue, not confined to a specific subsystem.

Finally, it is worth considering that adults who stutter also show a reduced speech motor response in feedback adaptation studies, where the altered feedback is predictable and maintained over consecutive trials^65^. It would be of interest to examine the adaptation results in the same detail as in the present study to determine whether timing variability is contributing to the observed differences in longer-term adaptive learning. Contrary to adults, however, children who stutter do not exhibit such reduced speech motor adaptation relative to controls^65^, which may reflect a more tolerant system of auditory-motor coupling in younger talkers. It would be interesting to see whether children who stutter resemble the adults tested in this study in their compensation to short-term perturbations. Comparing children and adults would allow us to better understand how developmental considerations impact the manifestations of the disorder, and take a step closer to knowing what makes developmental stuttering persist or resolve.

## Electronic supplementary material

Supplementary Information

Dataset 1

## Data Availability

In order to protect the privacy of participants, the raw vocal and video data is not publicly available. The processed data generated during the current study are available as supplementary material.
