# Supplementary material for: Timing variability of sensorimotor integration during vocalization in individuals who stutter
Source: Sci Rep. 2018 Nov 5;8:16340. doi: 10.1038/s41598-018-34517-1 (PMC6218511; doi:10.1038/s41598-018-34517-1)
Supplement: Supplementary file 1 — Supplementary Information [file 41598_2018_34517_MOESM1_ESM.pdf]

Supplementary material for *Timing variability of sensorimotor integration during vocalization in individuals who stutter*

Anastasia G. Sares*[1]
Mickael Deroche[1]
Douglas M. Shiller[2]
Vincent L. Gracco[1,3]

* Corresponding author: anastasia.sares@mail.mcgill.ca

1. Integrated Program in Neuroscience and School of Communication Sciences and Disorders, McGill University, Montréal, QC, Canada
2. School of Speech-Language Pathology & Audiology, Université de Montréal, Montréal, QC, Canada
3. Haskins Laboratories, New Haven, CT, USA

Self-ratings versus Speech-Language Pathologist ratings

• In the main article, we used self-evaluations of stuttering severity. The values of the correlations using the SLP ratings (on correctly-classified AS) were:

-percent opposing trials: $r^2 = 0.11$, $p = 0.274$
-onset time variability: $r^2 = 0.07$, $p = 0.385$
-peak time variability: $r^2 = 0.22$, $p = 0.105$

Our present view is that the SLP ratings were not accurate enough to capture the relevant variance in the data with the smaller sample. Note that our SLP was nevertheless highly trained and specialized in stuttering, emphasizing that 10-minute video clips may simply not be enough to extract a reliable assessment, and this is why we had decided to focus on self-ratings of stuttering severity, which, though subjective, draw from years of speaking experience rather than 10 minutes in the lab. References 48 & 49 from the main article give more information on the interchangeability of self-ratings with SLP ratings:

48. O'Brian, S., Packman, A., & Onslow, M. Self-rating of stuttering severity as a clinical tool. *Am J Speech Lang Pathol*. **13(3),** 219–26 (2004). http://doi.org/10.1044/1058-0360(2004/023)
49. Karimi, H., Jones, M., O'Brian, S., & Onslow, M. Clinician percent syllables stuttered, clinician severity ratings and speaker severity ratings: Are they interchangeable? *Int J Lang Commun Disord.* **49(3),** 364–368 (2014). http://doi.org/10.1111/1460-6984.12069

• The following figure includes all 19 participants, and uses the SLP severity scores:

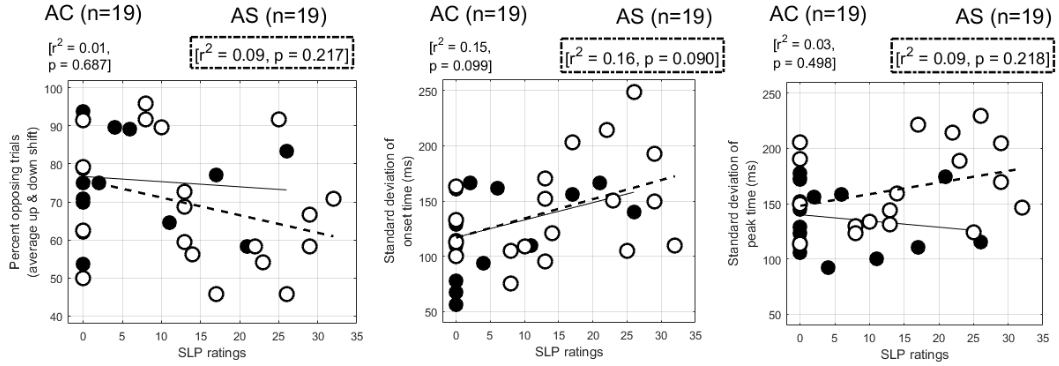

• Here, the lower correlation values seem to be due to some individuals who stutter with severity scores of zero, despite the fact that they have a low percentage of opposing trials and a higher peak time variability. It could be that these were the participants whose stuttering was not captured well during the 10-minute video. As stuttering can vary in different situations, this is not surprising.

• If we instead take all participants with an SLP score above zero, regardless of their eventual group classification, we see that even some of the behavior of controls can be accounted for by these ratings:

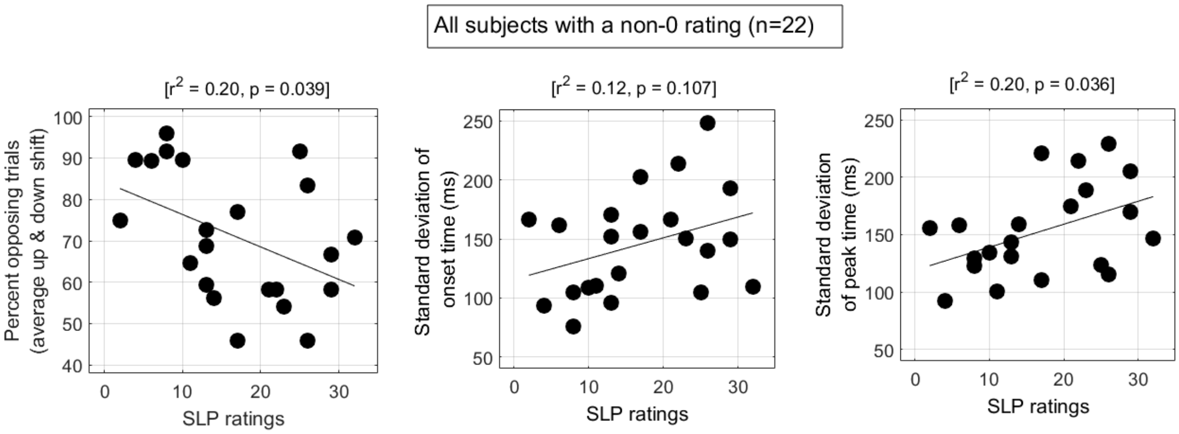

Whether this relationship in controls is related to developmental stuttering or other speech-motor characteristics is not clear.

• In sum, the self-ratings obtained in this experiment were better able to predict sensorimotor behaviors. SLP ratings could be more reliable, perhaps, if the SLP had a more representative video clip for each participant. In retrospect, we would recommend a greater amount of free monologue, or any type of speech sample that encourages more complex sentence structures.
